# Environmentally Toxic Solid Nanoparticles in Noradrenergic and Dopaminergic Nuclei and Cerebellum of Metropolitan Mexico City Children and Young Adults with Neural Quadruple Misfolded Protein Pathologies and High Exposures to Nano Particulate Matter

**DOI:** 10.3390/toxics10040164

**Published:** 2022-03-29

**Authors:** Lilian Calderón-Garcidueñas, Angélica González-Maciel, Rafael Reynoso-Robles, Héctor G. Silva-Pereyra, Ricardo Torres-Jardón, Rafael Brito-Aguilar, Alberto Ayala, Elijah W. Stommel, Ricardo Delgado-Chávez

**Affiliations:** 1 College of Health, The University of Montana, Missoula, MT 59812, USA; 2 School of Health Sciences, Universidad del Valle de Mexico, Mexico City 14370, Mexico; rafael.brito@uvmnet.edu; 3 Instituto Nacional de Pediatría, Mexico City 04530, Mexico; agonzalezmaciel@yahoo.com (A.G.-M.); reynosoraf@yahoo.com (R.R.-R.); 4 Instituto Potosino de Investigación Científica y Tecnológica A.C., San Luis Potosí 78216, Mexico; hector.silva@ipicyt.edu.mx; 5 Instituto de Ciencias de la Atmósfera y Cambio Climático, Universidad Nacional Autónoma de Mexico, Mexico City 04510, Mexico; rtorres@unam.mx (R.T.-J.); ridech@live.com (R.D.-C.); 6 Sacramento Metropolitan Air Quality Management District, Sacramento, CA 95814, USA; aayala@airquality.org; 7 Department of Mechanical and Aerospace Engineering, West Virginia University, Morgantown, WV 26506, USA; 8 Department of Neurology, Geisel School of Medicine at Dartmouth, Hanover, NH 03755, USA; elijah.w.stommel@hitchcock.org

**Keywords:** air pollution, Alzheimer’s disease, brain, cerebellar endothelial erythrophagocytosis, endothelial dysfunction, locus coeruleus, solid nanoparticles, nuclear pore complexes, Parkinson’s disease, TDP-43

## Abstract

Quadruple aberrant hyperphosphorylated tau, beta-amyloid, α-synuclein and TDP-43 neuropathology and metal solid nanoparticles (NPs) are documented in the brains of children and young adults exposed to Metropolitan Mexico City (MMC) pollution. We investigated environmental NPs reaching noradrenergic and dopaminergic nuclei and the cerebellum and their associated ultrastructural alterations. Here, we identify NPs in the locus coeruleus (LC), substantia nigrae (SN) and cerebellum by transmission electron microscopy (TEM) and energy-dispersive X-ray spectrometry (EDX) in 197 samples from 179 MMC residents, aged 25.9 ± 9.2 years and seven older adults aged 63 ± 14.5 years. Fe, Ti, Hg, W, Al and Zn spherical and acicular NPs were identified in the SN, LC and cerebellar neural and vascular mitochondria, endoplasmic reticulum, Golgi, neuromelanin, heterochromatin and nuclear pore complexes (NPCs) along with early and progressive neurovascular damage and cerebellar endothelial erythrophagocytosis. Strikingly, FeNPs 4 ± 1 nm and Hg NPs 8 ± 2 nm were seen predominantly in the LC and SN. Nanoparticles could serve as a common denominator for misfolded proteins and could play a role in altering and obstructing NPCs. The NPs/carbon monoxide correlation is potentially useful for evaluating early neurodegeneration risk in urbanites. Early life NP exposures pose high risk to brains for development of lethal neurologic outcomes. NP emissions sources ought to be clearly recognized, regulated, and monitored; future generations are at stake.

## 1. Introduction

Air pollution exposure is a well-established risk factor for adverse respiratory outcomes, stroke and cardiovascular morbidity and mortality [1,2,3]. Common neurodegenerative diseases affecting millions of people across the world have also been associated with air pollution [4,5,6,7,8,9,10,11,12,13,14]. Forensic autopsies in children, teenagers and young adult residents in Metropolitan Mexico City (MMC) exhibited quadruple aberrant protein pathologies: Alzheimer’s disease (AD), Parkinson’s disease (PD) and TAR DNA-binding protein 43 abnormalities [10,12]. In an autopsy cohort of 203 MMC previously clinically healthy individuals (age 25.36 ± 9.23 years), all, except a 22 year-old female with a TLR4 Asp299Gly polymorphism, showed AD neuropathology hallmarks, as defined by the presence of hyperphosphorylated tau protein (p-τ) and amyloid ß 17–24 [10]. We have reported that AD and PD neuropathology hallmarks overlap in young (≤40 years) MMC residents, starting in childhood and accompanied by olfactory bulb, auditory and vestibular nuclei pathologies, and a marked dysmorphology in the ventral cochlear nucleus and the superior olivary complex [15,16,17]. Hyperphosphorylated tau in the substantia nigrae pars compacta (SNpc) of children and young adults is documented in association with extensive degranulation of dopaminergic neurons, extracellular neuromelanin (NM) and macrophages phagocytosing NM. The major aberrant protein in MMC residents’ SNpc is hyperphosphorylated tau [12]. Given that young adults have gait and equilibrium abnormalities [18] and the cerebellum contains the highest concentrations of magnetic nanoparticles (NPs) in MMC young residents [12], we were interested in their NP cerebellar composition. Here, we investigated the association between key aberrant proteins p-τ and α-synuclein in primary dopaminergic and noradrenergic nuclei and the cerebellum and the presence of targeted NP elemental profiles. Damage to the SNpc, LC, and cerebellum during pediatric ages significantly alters key networks modulating autonomic function, arousal, motor control, cognition and emotions [19,20,21,22,23,24,25,26,27,28,29,30,31]. Critically important for this work is prior crucial research related to p-τ pathology as an initiating factor in sporadic AD, Dr. H. Braak and colleagues’ work describing the beginning of AD in the locus coeruleus, LC pathology indicating a continuum of Lewy body dementia, p-τ immunoreactivity identified in Bergmann glia, plus the key observation LC and SN neurons accumulate toxic metals [32,33,34,35,36,37,38].

We have three primary aims for the forensic 197 LC, SNpc and cerebellar samples from 179 cases previously examined for p-τ and α-synuclein, aged 25.9 ± 9.26 years plus seven subjects ≥41 years (63 ± 14.5 years): 1. To study by transmission electron microscopy (TEM) the ultrastructural alterations in the targeted regions and to identify NPs in neural and vascular cells’ organelles; 2. To image and analyze NPs within the sub-cellular environment at near-atomic resolution, using high resolution scanning and transmission electron microscopy (HRSTEM) and EDX and 3. Given that our proxy for fine particulate matter (PM_2.5_) exposures does not accurately represent NP exposures [6], mostly originating from combustion traffic processes and atmospheric new particle formation, and considering carbon monoxide (CO) is a key tracer of older vehicle emissions, we have assumed multiple non-linear correlations of simultaneous NP, PM_2.5_ and CO measurements carried out in several road sites in MMC that might provide an estimate of the expected historical patterns of UFP exposures in targeted areas. This approach could be helpful to evaluate the detrimental effects of NPs in selected populations based on the principle that early NP identification, composition, size, and subcellular location defines the potential NP toxicity and their biological impact upon the development of AD and PD. Our working hypothesis posits that the physicochemical characteristics of solid NPs in targeted brain regions encompass biologically plausible common denominators associated with aberrant protein accumulation and severe oxidative stress in the setting of lifetime exposures to air pollution, starting in utero [7] and continuing in pediatric and young adulthood ages. These NPs come from anthropogenic sources that should be readily and promptly controllable by the pertinent environmental protection authorities.

## 2. Materials and Methods

### 2.1. Study Area Air Quality

**Figure 1:**
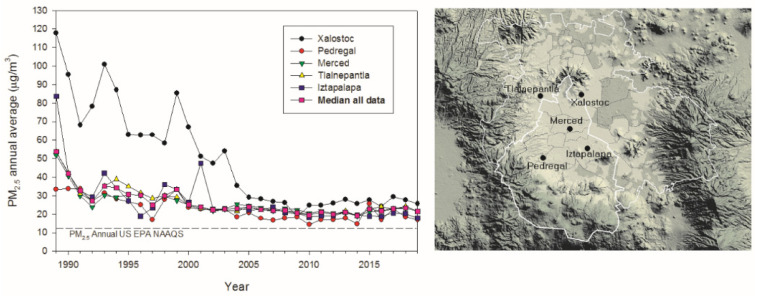
Time series trend of annual mean 24-h PM_2.5_ concentrations, averaged over 3 years, as well as the trend of the median for all data for five representative monitoring stations of the MMC from 1990 to April 2020 and their comparison with the US EPA NAAQS, and the map showing the location of the monitoring stations. Data were evaluated from measurements reported by the manual PM network of the Secretaría del Medio Ambiente del Gobierno de la Ciudad de México (SEDEMA) under a 6-day sampling schedule. The central white contour represents the limits of Mexico City and the secondary contour shows the MMC boundary. Source of data and figures: http://www.aire.cdmx.gob.mx/default.php# accessed on 13 January 2022 http://www.aire.cdmx.gob.mx/images/monitoreo/mapa-parametros/Mapa_relieve.jpg accessed on 14 January 2022.

The MMC area covers ~7585 km^2^ and is located on an elevated basin 2240 m above sea level surrounded by mountain ridges on three sides (Figure 1). MMC has a population of ~21.8 million people. Emissions from ~five million vehicles operating sub-optimally due to the altitude, over 50,000 industries, leaks from millions of portable house LP gas cylinders and gasoline distribution pipelines, extensive use of industrial, commercial and household solvents, and evaporative emissions of oil combine with high solar radiation and poor ventilation to produce a severe air pollution problem with a strong oxidizing capacity [39,40]. MMC residents have lifelong exposures to high levels of primary particulate matter air pollution from traffic, industry-related emissions, and resuspension of dust, as well as from secondary aerosols formed from atmospheric chemical reactions of abundant precursors [39,41,42]. Chronic exposure levels to PM_2.5_ are above World Health Organization (WHO) guidelines and the US Environmental Protection Agency (USEPA) standards. The USEPA annual PM_2.5_ standard of 12 µg/m^3^ has been exceeded across the MMC area for the last 30 years (Figure 1).

Typically, the highest PM_2.5_ concentrations occur in the NE sector (Xalostoc), associated with intense industrial and heavy-duty diesel traffic, and decrease towards the SW (Pedregal) residential area [43]. Exposures to ozone (O_3_) concentrations have been above the USEPA standard (annual fourth-highest daily maximum 8-h concentration, averaged over 3 years) all year long since the 1980s [44]. Due to the nature of O_3_ formation and diurnal winds, the highest levels are observed in SWMMC [41,42]. Other criteria pollutants, including nitrogen dioxide, sulfur dioxide, carbon monoxide and lead, displayed elevated levels prior to 2005, but they have shown a continuous decrease at or below the respective current EPA standards in the last 15 years. PM_10_ has followed a similar trend as per PM_2.5_ with levels exceeding the USEPA air quality standards [39,43]. MMC residents have been exposed to high levels of primary fine and as well as secondary air pollutants, including secondary inorganic and organic aerosols and O_3_ concentrations with frequent maxima levels above the United States National Air Ambient Quality Standards (NAAQS) all year round, at least during the last three decades [42,43,44,45,46,47]. High levels of black carbon (BC), polycyclic aromatic hydrocarbons (PAHs), semi-volatile organic compounds from incomplete combustion of carbonaceous fuels such as gasoline and diesel, as well as metals from fuel and lubricating oils, and engine, brake, and tire wear have been historically found in MMC PM_2.5_ [48,49,50,51,52,53,54,55,56,57,58,59,60]. The most abundant metals and metalloids in PM_2.5_ in the last decade have followed the approximate trend: Si > Fe > Al > Ti > Ca > Sr > Zn > Cu > Pb > Cr > Mn [59,60]. Important Hg concentrations in PM_2.5_ are more abundant in NEMMC, where large industrial facilities including agrochemical, pigments, dyes, pulp and paper, and manufacturing industries, are located [61]. Commuting in any of the urban transport modes available in the urban area is associated with high NP exposures [55]. Moreover, exposures in the Mexico City subway led to high indoor PM_2.5_ between 34 and 93 μg m^−3^ with elevated concentrations of Fe, Cu, Ni, Cr and Mn; equivalent PAH concentrations ranging from 19 to 41 ng m^−3^; and NPs with average size 38.5 ± 15.9 nm reaching up to 50,300 ± 10,600 (# cm^−3^) [55]. Metals in the underground subway are the result of friction, brake wear, and sparking from rail grinding [55,62].

Although NPs may have a greater potential for inducing adverse health effects than larger particles, they are currently neither monitored nor regulated. The few studies historically performed in MMC have shown that outdoor ultrafine particles (UFPs) in the proximity to high traffic avenues could reach high levels [53,54,55,63,64]. It is important to remember that in general there is no direct correlation between NPs and PM_2.5_ concentrations in ambient air. As in any urban atmosphere, UFPs in MMC account for the majority of the number of particles, but they are almost negligible in mass concentrations compared to particles greater than 100 nm [64,65]. As combustion is considered the main source of UFPs and given their physicochemical nature, their number concentrations (PNCs) are strongly dependent on the distance from high traffic avenues, with larger concentrations close to the roadside and lower numbers away from the vehicular emissions due to dispersion and possible other effects [66]. The majority (~90%) of UFPs consists of volatile and semi-volatile, carbon-bearing phases, while the primary, solid combustion vehicle-derived NPs are enriched in highly reactive transition metals, especially Fe, Cu, Mn, Ti, Cr, Ni, V, Pb and Zn [67,68]. Metal-bearing and free-floating NPs are abundant in the MMC atmosphere [69]. More than 60% of such NPs collected by aircraft and analyzed (*n* = 572) by TEM contained Fe, Zn and/or Pb [69]. The abundance of metals in NPs follow this order: Fe > Zn > Pb > Mn > Hg > Sn > Ni > Cr > Ti > V > Ag [69]. Key to this work, Hg has been identified in ~21% of free-floating NP samples [69]. Moffett et al. [70] found Zn- and Pb-bearing particles ranging in size from 0.2 to 2.0 μm in MMC aerosols, the typical emissions source being road traffic. NPs are also produced by vehicle brake wear [71]. MMC residents, including children and pregnant women, have been inevitably exposed to outdoor elevated UFPs and NPs rich in metals and PAHs [6,7,39,40,41,42,43,44,45,46,47,48,49,50,51,52,53,54,55,56,57,58,59,60,61,62,63,64,65,66,67,68,69,70].

### 2.2. Study Design and Brain Samples

We previously examined 203 MMC forensic autopsies in subjects dying in accidents, homicides and suicides with an average age of 25.3 ± 9.2 years and evaluated each case with diagnostic antibodies for hyperphosphorylated tau, beta amyloid and alpha-synuclein [10,12,14,17]. In this revisited cohort of lifelong 179 MMC residents with an average age of 25.9 ± 9.2 years and seven additional subjects ≥41 years old (average age of 63 ± 14.5 years, range 45–85 years), we had 78 cerebellar, 62 substantia nigrae pars compacta and 57 locus coeruleus samples available. The 23 children included had an average age of 13.01 ± 4.9 years and all had SNpc, LC and cerebellar samples. Autopsies were performed 3.7 ± 1.7 h after death and included subjects with no major pathological findings, other than the acute cause of death [10,12,16]. Examination of autopsy materials was approved by the Forensic Institute in Mexico City and autopsies were performed in a five-year period between 2004 and 2008. Brains were examined macroscopically; sections were selected for light and electron microscopy. Brainstems were sectioned from the midbrain at the level of the superior colliculi to the lower medulla, with an average of 13.6 ± 4.4 paraffin blocks and 48.7 ± 12.0 slides per individual paraffin block. Left cerebellar hemisphere samples were taken. Paraffin embedded tissue was sectioned at a thickness of 7 μm and stained with hematoxylin and eosin (HE). Immunohistochemistry (IHC) was performed on serial sections as previously described [10]. Antibodies included: PHF-tau8 phosphorylated at Ser199–202-Thr205 (Innogenetics, Ghent, Belgium, AT-8 1:1000) and α-synuclein phosphorylated at Ser-129, LB509 (In Vitrogen, Carlsbad, CA, USA, 1:1000).

### 2.3. Transmission Electron Microscopy (TEM), High Resolution Scanning and Transmission Electron Microscopy (HRSTEM) and Energy-Dispersive X-ray Spectrometry (EDX) Studies

Studies were performed using three mm blocks. Samples were cut with ceramic knives and handled with plastic forceps, free from metal contamination. Sections were fixed in 2% paraformaldehyde and 2% glutaraldehyde in sodium phosphate buffer for TEM and EDX studies. The focus of the SN, LC, and cerebellum evaluation using conventional TEM (JEOL-1011, Osaka, Japan, operated at 80 kV) was to document the integrity of the neurovascular unit (NVU) and to define the location of the electrodense NPs present within target organelles of different cell types. For identification and elemental analysis of NPs, we additionally cut 70 nm unstained tissue sections and placed them on Ni mesh grids. A 300 kV FEG FEI TECNAI F30 TEM tuned to a 100 kV acceleration beam and an 11 spot size was also utilized for examining the samples. We analyzed all samples blind to case and grids/tissue sections and grid areas were randomly selected and methodically scanned. Analysis was conducted to identify NP elemental composition and ultramicroscopic structure of neural and vascular cells and organelles. The organelle NP location and their elemental content were the focus of this study.

## 3. Results

### 3.1. Air Pollution

While the historical exposure to PM_2.5_ can be tracked from ambient air monitoring data records, the evaluation of long-term exposures to NPs depends on direct measurements of nanoparticle number concentration (PNC). Since there were just a few studies on NPs in MMC, we decided to approximate the PNC of UFPs through the correlation that NPs have with other pollutants that have been identified as associated with UFPs [72]. Our approach was based on the fact that UFPs originate predominantly from traffic-related internal combustion processes in urban areas and to a lesser extent, atmospheric new particle formation processes [72]. Therefore, considering that carbon monoxide (CO) is a main tracer of vehicular emissions, and that UFPs are a very small fraction of the PM_2.5_ mass concentration, we assume that multiple non-linear correlations between available data from simultaneous measurements of NPs, PM_2.5_ and CO carried out in several road sites in MMC can provide us with an estimate of the expected historical patterns of UFPs in this urban area. A number of studies have reported a strong correlation between NPs and CO and a weak-to-moderate association between UFPs and PM_2.5_ [53,64,65,66,67,68,69,70,71,72,73,74]. We used geometric means of measurements of PNC, PM_2.5_ and PAH reported by Velasco et al. [55] for 15 outdoor roadside locations in the MMC center area carried out during 2017 to obtain the non-linear regression equation. Then, we used the equation to estimate the trend of annual PNC values from the annual medians of measured PM_2.5_ and CO available from the official monitoring network in MMC from 1989 to 2019. The PNC reported by Velasco et al. [55] corresponded to an estimated particle size of <50 nm. These data were combined with CO values estimated from the correlation of PAH and CO obtained by Ladino et al. [51] from independent measurements of PAH with a similar instrument as that used by Velasco et al. [55], as well as with a more sensitive CO monitor, in a representative background urban site in SWMMC during the same year. The resulting multiple non-linear correlation equation was:

PNC = −13,335.17 + (811.377 × PM_2.5_) + (58.24 × CO): R^2^ = 0.58 (1)

where: 

PNC = Particle number concentration of NPs in # cm^−3^;

PM_2.5_ = PM fine fraction in μg m^−3^;

CO = Carbon monoxide in μg m^−3^.

Equation (1) was used to estimate the approximate annual trend of the PNC for the period 1989–2019 using the annual medians of PM_2.5_ and CO mass concentrations derived from data reported by the official MMC monitoring network. While CO data were available for the whole study period, data on PM_2.5_ were obtained from a combination of direct measurements and correlations between PM_2.5_ and PM_10_. Due to systematic PM_2.5_ measurements not being available until 2004, fine particle concentrations for each year of the period 1989 to 2004 were estimated from PM_10_ measurements performed in the same monitoring for that period of time using the average of the slopes of the linear regression analysis of the 24-hr means of PM_10_ and PM_2.5_ for each year and site from 2004 to 2008 according to:

(2) $${\mathrm{PM}}_{2.5}{}_{\left(\mathrm{estimated}\right)}={\mathrm{PM}}_{10}{}_{\left(\mathrm{measured}\right)}\times {\left[\frac{{\mathrm{PM}}_{2.5}}{{\mathrm{PM}}_{10}}\right]}_{\mathrm{slope}}$$

Since neither PM_10_ nor CO data were available before 1989, we assumed the estimated PM_2.5_ level for previous years was equivalent to the value calculated for the year 1989.

**Figure 2:**
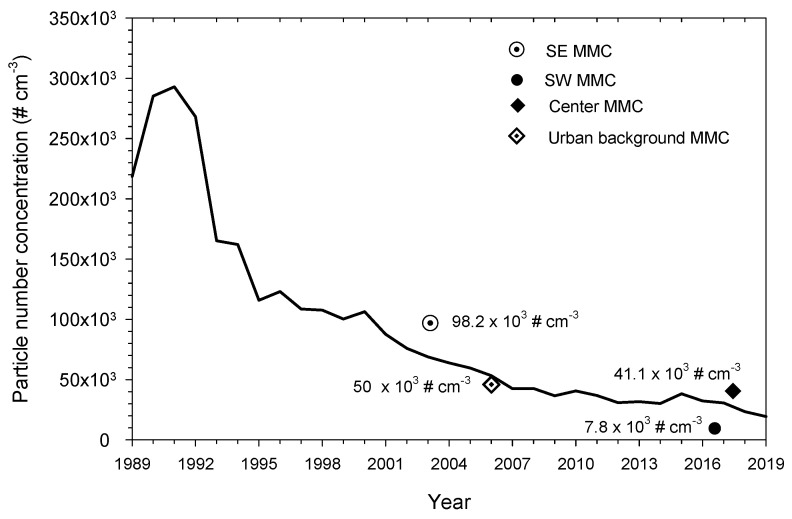
Annual trends of PNC of NPs estimated from the medians of annual measured and estimated PM_2.5_ and CO registered by all monitoring stations of the MMC from 1989 to 2019. The symbols in the figure correspond to the median PNC and the date of measurement reported by: ⊙ Dunn et. al. [53], commercial with median industry and heavy traffic site, size of measured NPs between 3–15 nm; ● Caudillo et al., [54] residential with low traffic site, size of measured NPs between 20–100 nm; ◆ Velasco et al., [55] commercial with moderate to heavy traffic, size of measured NPs < 50 nm; ◈ Kleinman et al. [64], PNC urban background estimated from the extrapolation down to surface of the average NPs with sizes < 100 nm measured by aircraft across the MMC at an altitude average level of 350 m above surface. Source of data was obtained at: http://www.aire.cdmx.gob.mx/default.php# accessed on 13 January 2022.

A very important assumption for this approach is that the resulting non-linear regression equation obtained with 2017 data represents the expected behavior of PNC for previous years. Figure 2 shows the estimated trend.

The estimated PNC trend coincides with the PNC values reported for the short-term studies shown in Figure 2, which provides reasonable confidence of the results. The high PNC estimated in the 1990s is in parallel with the very high levels of other criteria air pollutants registered in North America [39]. The estimated trend for PNC follows the downward trend of PM_2.5_ before 2002, but while the PNC kept a slow decrement with time, the PM_2.5_ annual medians barely show a downward trend. The trend of PNC in the last two decades suggests that exposures to very high NP concentrations are very important for MMC residents born before 2002, including the great majority of subjects in this study. MMC residents born after 2002 have been continuously exposed to PNC of NP levels close to the overall average of 44,000 cm^−3^ recorded for 40 urban areas of Asia, Europe, North America and Australia [66]. It is key to keep in mind that MMC residents are exposed to NPs with high metal content and given the effect of altitude (2240 m above sea level), they have a higher breathing rate [75], such that the NPs inhaled by a MMC resident are higher than other polluted cities in the world documented so far [66].

### 3.2. Light and Electron Microscopy in Cerebellum, Substantia Nigrae and Locus Coeruleus

The pτ and αSyn status of the substantia nigrae and locus coeruleus were accomplished in previous studies [10,12,16,17]. A summary of IHC results is shown in Table 1. We have documented substantia nigrae pτ in 54.3% of samples (*n* = 100) and α-Syn in 22.8% (*n* = 42), while in locus coeruleus pτ was documented in 44.5% (*n* = 82) and α-Syn in 24.8% (*n* = 44). Individuals with LC α-Syn were the oldest with an average age of 27.9 ± 8.2 years. Cerebellar samples were negative for pτ and/or α-Syn.

**Figure 3:**
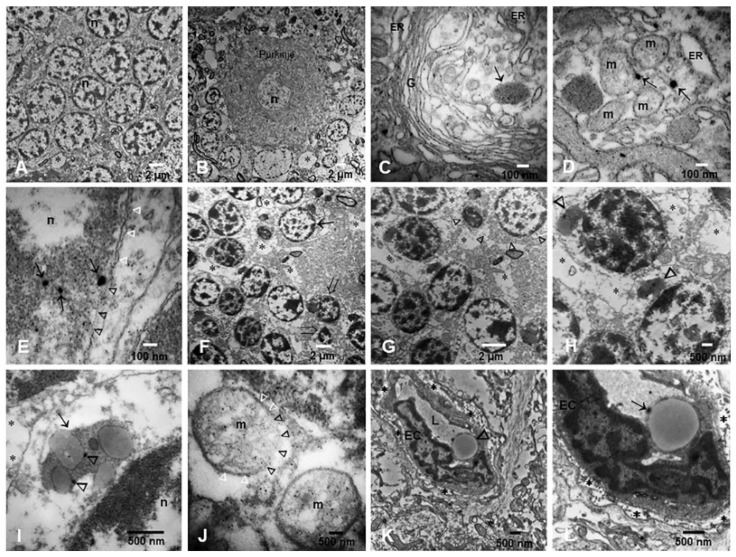
TEM in cerebellum. (**A**) Granular layer neurons in a 3 year-old male. A few, vacuolar (*) spaces are seen between neurons. (**B**) One Purkinje cell is surrounded by glial and neuronal bodies. There is a significant number of vaculated spaces (*), particularly towards the molecular layer (upper third) where axons are identified. Same 3 year-old as (**A**). (**C**) Close-up of Golgi apparatus and dilated endoplasmic reticulum (ER) in a MMC 11 year-old child. Notice the lysosomal structure with nanoparticles (arrow). (**D**) Purkinje neuron cytoplasm exhibiting mitochondria with abnormal cristae and two distinct spherical NPs (arrows). The ER is markedly dilated. (**E**) Fourteen-year-old Purkinje cell nucleus with three spherical NPs (arrows) in the midst of heterochromatin. The double nuclear membrane is apparently intact along the white arrow heads but disappears under the black arrow heads. (**F**) Granular cell layer in a 36 year-old male shows a significant number of neuropil vacuolated areas (*) and a marked contrast between an apparently intact nucleus (arrow) and two nuclei with chromatin condensation, nuclear shrinkage, and the formation of apoptotic bodies (opened arrows). (**G**) A close-up of (**F**) shows the empty spaces between neurons (*) and the large lysosomal bodies (arrow heads) in the granular cells, seen also in (**H**). (**H**) These two neurons have already marked cytoplasmic changes with fragmented organelles and ill-defined cell membranes. (**I**) A conglomerate of lysosomal structures (arrow) with NPs (arrow heads). (**J**) In the same sample, two mitochondria with ill-defined membranes (black arrow heads) and apparently intact double membranes (white arrow heads). (**K**) Cerebellar blood vessel in a 45 year-old male with significant vacuolization and fragmentation of the perivascular neuropil (*). Notice the endothelial cell (EC) and the vessel lumen. (**L**) A close-up of the same vessel to show the erythrophagocytosis of the endothelium. The arrow points to a NP trapped between the erythrocyte engulfed by the endothelial cell and the endothelial coverture. The multiple *s point to the NVU breakdown.

The selection of SN and LC samples for TEM and EDX were based on immunohistochemistry (IHC) results, and we included 23 children aged 13.3 ± 4.1 years. Since we had no IHC pτ and/or α-Syn positive cerebellar cases, we selected 78 samples across the age ranges, including the 23 children. Cerebellar samples (Figure 3A–L) exhibited alterations in the neurovascular unit (NVU) starting in childhood (Figure 3A,B), worsening in individuals in the fourth decade of life (Figure 3F–H). NPs were identified in all neural and vascular cerebellar cells, from free particles to conglomerates in mitochondria, the Golgi apparatus, endoplasmic reticulum, endolysosomes and nuclei. Interestingly, nuclear-pore complexes in the nucleoplasm were occupied by NPs and discontinuation of the double nuclear membranes was observed frequently in children’s samples (Figure 3E). Granular cells exhibited significant structural damage with accumulation of NPs in abnormal mitochondria and lysosomal structures (Figure 3H–J). Significant vacuolization of the neuropil (*) was a striking observation in children (Figure 3B) and more severe in older individuals (Figure 3B,G,H). Vascular changes were outstanding, with endothelial phagocytosis of red blood cells (RBC) studded with NPs (Figure 3K,L).

**Figure 4:**
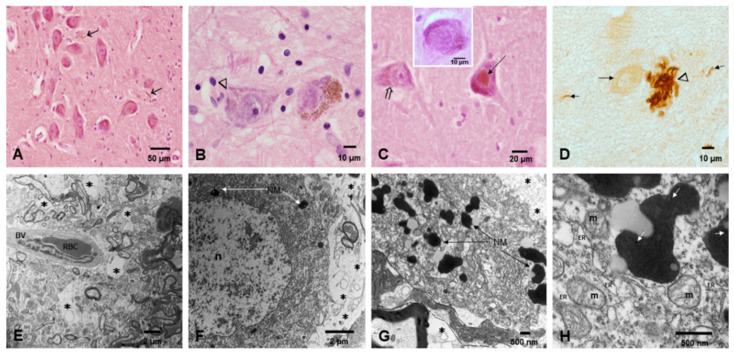
Substantia nigrae pars compacta SNpc. (**A**) Fourteen-year-old child with numerous + Pτ (arrows) in the SN pc. (**B**) Seventeen year-old male with contrasting SNsc neurons, the one on the left (arrow head) displays very little neuromelanin. (**C**) Eleven-year-old boy with intense nuclear Pτ (short arrow) alongside intracytoplasmic + Pτ (arrowhead)immunoreactivity. The insert shows a Lewybody like inclusion. (**D**) Pτ mature plaques (arrow head) and neurites (short arrows) are common in subjects ≥ 30 years with SNps neurons devoid of neuromelanin (long arrow). (**E**) TEM in a 3-year-old child with significant fragmentation and vacuolization (*) of the neuropil surrounding the blood vessel. (**F**) Same as (**E**), a neuron shows very few neuromelanin granules (white arrows), an apparently intact nucleus (n) and a significantly fragmented neuropil (*). (**G**) The amount of neuromelanin goes up in adults along significant neuropil fragmentation (*). (**H**). Neuromelanin granules with NPs (white arrows), abnormal mitochondria (M) and markedly dilated endoplasmic reticulum (ER).

Positive pτ neurites and nuclei were identified in children’s SNpc (Figure 4A–C). In brains of teens, an occasional Lewy body was observed in the SNpc, but pτ neurites surrounded by extracellular neuromelanin were more prominent. The brains of individuals ≥30 years showed tau plaques in the SNpc (Figure 4D). Breakdown of the NVU in the SN was an early finding in toddlers (Figure 4E) and vacuolization of the neuropil and structural changes in SN neurons worsened with age (Figure 4F–H). Numerous NPs were seen in neuromelanin and abnormal mitochondria (Figure 4G,H).

**Figure 5:**
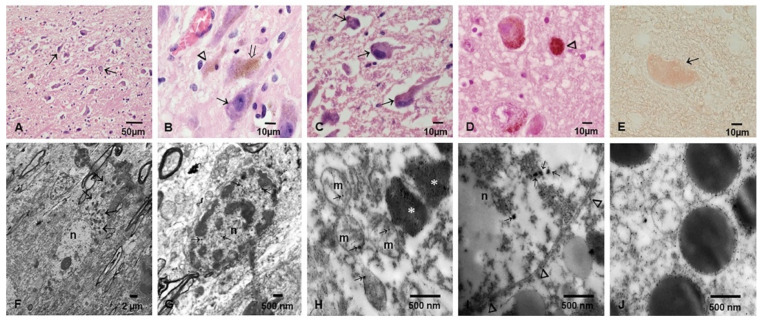
Locus coeruleus light and electron microscopy. (**A**) LC in a 17 year-old male, neurons are marked with short arrows (H&E). (**B**) A 14-year-old girl; notice the contrast between an apparently intact cell (open arrow), a neuron totally devoid of neuromelanin (short arrow) and a macrophage packed with NM (arrowhead). (**C**) A close-up of A to show the rounded appearance of the ill-defined neurons (short arrows). (**D**). A 27-year-old male with macrophages packed with NM (arrowhead) alongside intact and degranulated neurons. (**E**) Fourteen-year-old girl with α-synuclein + neuron (red product, no contrast staining). (**F**) LC neuron with numerous NM granules (short arrows) in a 33-year-old male. (**G**) A close-up of a LC neuron in a 11-year-old child with numerous NPs in the nucleus (n) (short arrows) and very ill-defined cytoplasmic borders. (**H**) Same 11-year-old, several lysosomal bodies (*) with NPs and every single mitochondrion with fragmented cristae and NPs (short arrows). (**I**) Same as (**G**,**H**), numerous NPs inside the nucleus (short arrows) and NPs going through the nuclear pore complexes (arrow heads). (**J**) Significant number of dark lysosomal structures decorated with NPs and surrounded by ill-defined organelles and NPs.

Significant degranulation of LC neurons and macrophages containing neuromelanin was recorded in children and young adults (Figure 5A–D) with accumulation of pτ and α-synuclein (Figure 5E). TEM showed neuropil changes around LC neurons and structural alterations in neuromelanin, mitochondria and numerous NPs free in the neuronal cytoplasm, as well as in NM, lysosomes and mitochondria (Figure 5G–L). Strikingly, nuclear-pore complexes in the nucleoplasm were occupied by NPs, which were identified along the double nuclear membranes (Figure 5I).

### 3.3. EDX

**Figure 6:**
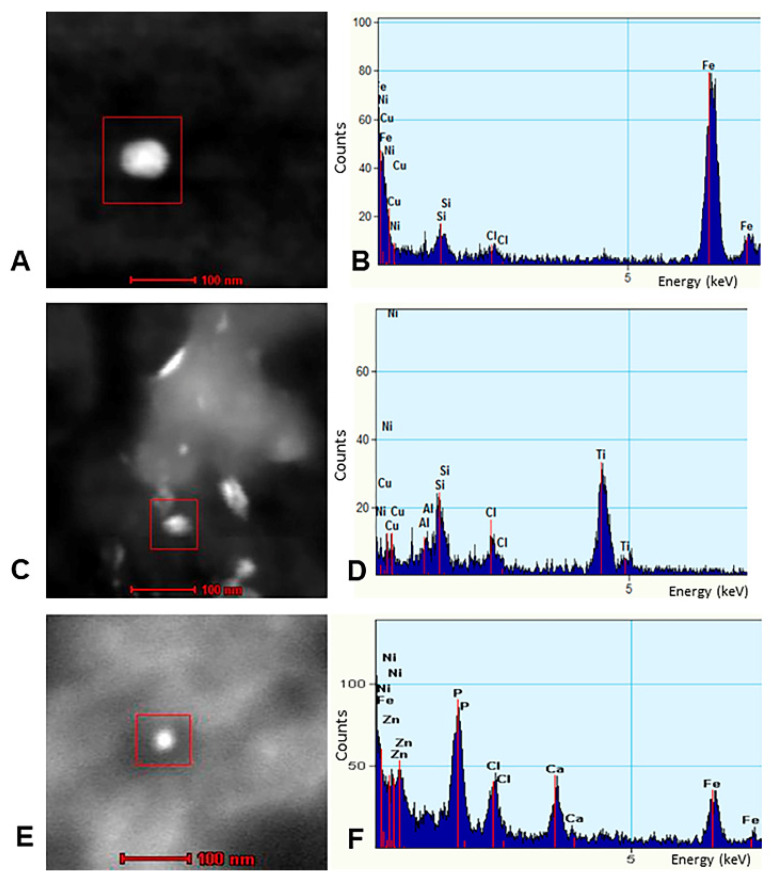
NP characterization by TEM and EDX in human granular cell layer, cerebellum. (**A**) One spherical NP is enclosed by a red square. (**B**) EDX identifies Fe as the main element in the NP. (**C**,**D**) Typical crystal morphologies and fused surface textures of CFDNPs are co-associated with other reactive metals including Ti and Al. (**E**,**F**) A CFDNP particle displays Fe and Zn. Ni traces reflect the Ni TEM support grid.

**Figure 7:**
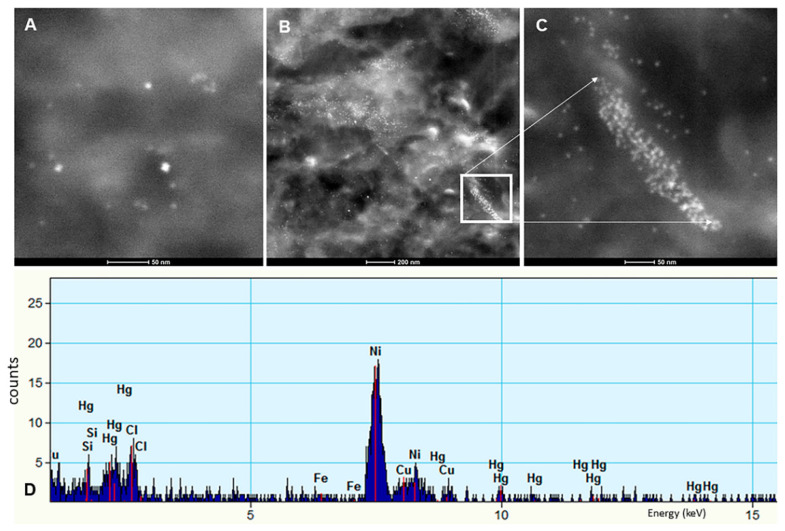
NP characterization by TEM and EDX in substantia nigrae. Different sizes and shapes of NPs are seen through the SNpc cells and the neuropil. (**A**) Three HgNPs are identified, (**B**) The predominant NPs are Fe and Hg in this sample. (**C**) In a close-up of a finger-like macrophage extension towards the neuropil (from square in (**B**)), we documented the accumulation of predominantly FeNPs. (**D**) Hg is the main element detected in another SNpc sample from a teen. Ni reflect the TEM support grid.

**Figure 8:**
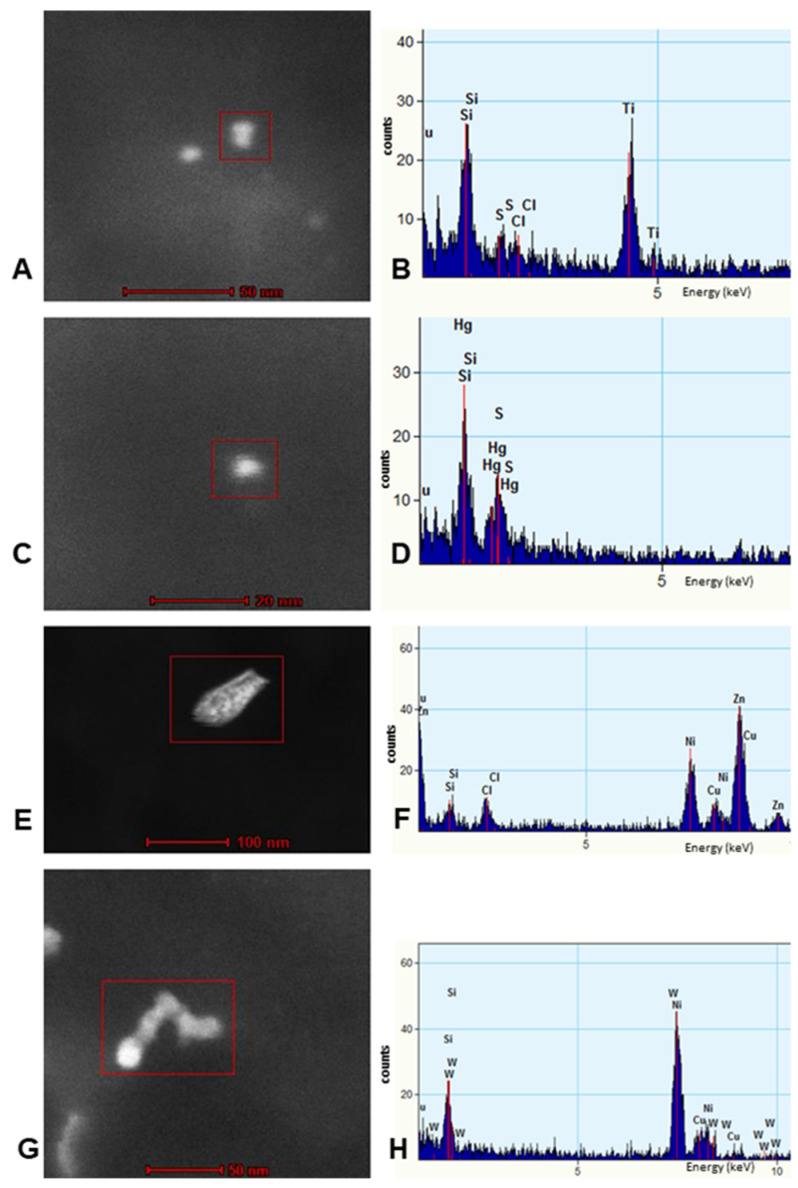
Locus coeruleus NP characterization by TEM and EDX. TiNPs were commonly identified throughout LC noradrenergic neurons (**A**,**B**). HgNPs were very common (**C**,**D**), while other metals such as W and Zn were also present (**E**–**H**). The Tungsten W NP in this picture comes from a 15-year-old girl (**G**) and measures 18 ± 2 nm wide and 77 ± 2 nm in length. Red squares included the NPs examined.

Individual particles in neural and vascular cells were analyzed by EDX analysis. Solid particles were rounded, acicular and electrodense and dominated by combustion- associated iron and transitional metals (Figure 6). We identified numerous FeNPs 4 ±1 nm, HgNPs 8 ± 2 nm and tungsten WNPs in the SN and LC of children and young adults (Figure 6, Figure 7 and Figure 8).

## 4. Discussion

Highly reactive environmental solid NPs in key noradrenergic and dopaminergic nuclei and cerebellum were identified in young urbanites, including toddlers. These young urbanites exhibit an early development of AD, PD and quadruple misfolded protein neurodegenerative pathologies and similarly exposed populations have extensive documentation of cognition deficits, rapid eye movement sleep behavior disorders, brainstem auditory evoked potentials and gait and equilibrium abnormalities [6,7,8,9,10,11,12,13,14,15,16,17,18].

The presence of environmental NPs—including engineered nanoparticles—causing significant early structural subcellular and NVU alterations raises the issue of the role of NPs in AD and PD development in MMC young residents. We identify a profile of metal solid NPs, including Fe, Ti, Hg, W, Al and Zn observed in the typical airborne PM_2.5_ pollution MMC mixtures, as a replication of anthropogenic and engineered NP sources influenced by local vehicular and subway exposures, open burning of trash, smelter and incineration emissions and engineered NP exposures [47,53,54,55,59,61,62,69,70,72]. We posit that there is the potential for greater effects if it were possible to attribute damage to volatile and semi-volatile NPs, which are invariably present in the mix of pollution or absorbed onto the NPs identified in the particle phase.

The detrimental NP neural effects including early damage to the NVU are of deep concern. Specifically, the molecular heterogeneity of cerebrovascular cells likely contributes to their vulnerability to NPs and the resulting damage in targeted vascular neurons and glial cells [76]. The in vitro work of Coccini et al. [77] is relevant; NP uptake resulted in a reduction in neuronal differentiation with downregulation of β-tubulin III, microtubule-associated protein 2, enolase and nestin. Mehrbeheshti et al., [78] used aluminum NPs by oral gavage in mice and induced dose dependent memory deficits coinciding with an increase in hippocampal phosphorylated p38 and cleaved caspase 3, while Mortensen et al. [79] showed the impact of TiO2 NPs on neurobehavioral performance.

It has been shown that the substantia nigra pars compacta and locus coeruleus accumulate NPs with highly reactive metals in neuromelanin (NM) and endosomal structures [12,14] and display significant age changes, including free neuropil NM and increased microglial phagocytic activity [80]. Moreover, Pamphlett et al. [37] showed that LC neurons accumulate Hg selectively and strikingly; 47% of the 190 adults included in their study (≥20 years) also had cadmium, silver, lead, iron, and nickel detected by autometallography (AMG™). Pamphlett and Bishop [36] showed the presence of Hg in substantia nigra, motor cortex, striatum, thalamus, and cerebellar PD samples. The presence of Hg in neurons and oligodendrocytes in white and grey matter and Hg co-localized with Lewy bodies and neurites were also described [36]. In a striking resemblance, and in keeping with NP exposures in high altitude volcanic MMC [53,54,55,61,75], we show a LC/SN NP profile with Fe, Hg, W and Ti NPs in MMC children along with TEM structural changes and light microscopic extracellular neuromelanin. Key PD and AD hallmarks i.e., alpha synuclein and hyperphosphorylated tau are present in targeted regions, strongly suggesting early pediatric neurodegeneration go hand in hand with the presence of toxic <10 nm NPs. Outstandingly, the ≤10 nm NP sizes in the LC and SN could be relevant to the NP transportation mechanism described by Panja and Jana: a direct membrane-penetrating NP <10 nm offers high delivery efficiency, faster delivery kinetics, and minimal lysosomal degradation [81]. The authors showed arginine-terminated Au NPs <10 nm size enter via energy-independent direct membrane penetration, are directly transported into the cytosol within a minute and allow direct access to subcellular compartments [81]. The smaller NP sizes are also critical for prolonged blood circulation times and severe blood vessel damage [82,83]. Mercury exposures and transportation to vulnerable brain regions are a crucial subject for MMC residents: Hg, Cu, Zn, Fe, Al, Cd and Se cross the BBB, enter the brain and promote oxidative stress, mitochondrial dysfunction, and the formation of α-synuclein leading to dopaminergic neuronal damage [7,9,12,83,84]. Mercury is ubiquituous and naturally enriched in volcanic regions and anthropogenic activities release large amounts of Hg into the environment [61]. The chemical speciation of Hg determines its mobility and toxicity. Although methyl mercury is the predominant form of mercury during exposure, inorganic mercury slowly accumulates and resides in the brain for long periods of time and the fetal brain is particularly vulnerable to accumulated Hg concentrations, five to seven times higher than in maternal blood [7,36,37,85,86,87,88]. Damage to the BBB associated with methylmercury via active transport systems, mainly the l-type amino acid transporter, on endothelial cell membranes has been described in association with vascular dysfunction [89].

Tungsten (W) has been used extensively in MMC for decades, i.e., in the filaments of old light bulbs and fluorescent lighting; additional sources elsewhere include metal-working, mining and petroleum industries, military applications and medical devices [90,91]. W in LC and SN indeed is not a surprise. Remarkably, W increased the expression of IL-1β, tumour necrosis factor (TNF)-α, nerve growth factor (NGF) and brain-derived nerve factor (BDNF) along with the presence of nociceptive neurons at the endplates in intervertebral disc cells [90]. In the work of Chinde and Grover, the average size of spherical WO_3_ NPs and microparticles in TEM was 52 ± 2.97 nm and 5.7 ± 7.5μm, producing genotoxicity in blood, with the highest amount in the liver and the lowest in the brain of treated rats [91]. Adding to the complexity of the MMC atmospheric chemistry and the targeted nature of NPs, the NP literature is very helpful is pointing to the importance of the different NP concentrations, charges, corona characteristics, their zero-valent character, and their combined effects upon kinetics of β-amyloid and α-synuclein fibrillations [92,93,94,95,96,97,98]. Particularly relevant is the fact that seemingly harmless Si NPs [12,86] are as critical as Fe and TiO_2_ NPs [93,94,96,98] in altering protein fibrillation, producing oxidative stress and causing dysfunction of the ubiquitin-proteasome system and endolysosomal alterations [96,97].

The core issue is clearly stated: NPs can modulate protein aggregation and fibril formation in the context of amyloid diseases and are very critical to the problem of neural proteins: once the protein is altered—as in α-syn’s conformation changes—NP-bound α-syn initiates conformational changes in free α-syn [98,99,100,101]. The issue at stake in MMC residents relates to Braak and Del Tredeci et al.’s key papers on AD and PD development: “Abnormal tau lesions (non-argyrophilic pretangle material, argyrophilic neuropil threads, neurofibrillary tangles) in select types of neurons are crucial for the pathogenesis of sporadic Alzheimer’s disease. “Ongoing formation of these tau lesions persists into end-stage Alzheimer’s disease and is not subject to remission” [32]. “We hypothesize that tau pathology within select projection neurons with susceptible microenvironments can initiate sAD” [33]. “The earliest lesions (referring to PD) could develop at nonnigral (dopamine agonist nonresponsive) sites, where the surrounding environment is potentially hostile: the olfactory bulb and, possibly, the ENS” [102]. Moreover, the paper by Stopschinski et al. [38] is key for this work. The authors documented a progressive increase in tau seeding according to NFT stage: “seeding frequently preceded NFT pathology, e.g., in the basolateral subnucleus of the amygdala and the substantia nigra, pars compacta.”

We fully expected the presence of multiple aberrant proteins in MMC subjects highly exposed to NPs [10,12,14], in keeping with Karanth et al.’s [103] quadruple pathology described in elder subjects. Thus, the early location of NPs in key neuronal groups could be the common denominator for a number of neurodegenerative diseases associated with extensive free radical formation, magnetic and hypoxic effects, DNA damage and neural and vascular organelle damage [94,95,96,97,98,99,100,104,105,106,107,108,109,110]. At the crux of our findings are the identified NP elements: Fe, Ti, Hg, W, and Zn, pointing to the emission sources and the possibility of interventions to ameliorate exposures by the 21.8 million MMC residents. Diesel heavy-vehicles are a culprit and given their contribution to transition metals [67] and their lack of regulation in MMC, we strongly suggest that it should be a first task for emissions control, followed by Northern MMC industrial emissions [70]; the Hg sources identified in NEMMC [61], and last, but equally very important—because of the massive daily exposure to NPs—are the 17 million citizens’ commuting work week trips in MMC [55].

It is important to note that traveling in the MMC subway system exposed people to the highest NP concentrations, while walking and cycling resulted in the highest inhaled particles’ doses [55]. MMC residents using air-conditioned ride-hailing cars have the lowest exposure in regard to transport mode [55]. The presence of NPs in the LC, SNpc and cerebellum in young children and the early development of AD and PD obligates us to review the critical functions of the targeted brain regions related to arousal, attention, stress responses, central autonomic network, emotions, gait and equilibrium and to revisit key papers addressing these critical brain areas under physiological homeostatic conditions and in AD, PD and dementia with Lewy bodies (DLB) patients [111,112,113,114,115,116,117,118,119,120,121,122,123,124].

The presence of NPs in neuronal and glial cerebellar cells and our previous report the cerebellum has indeed the largest concentration of magnetite [12] is worth commenting upon. Damage to Purkinje cells will compromise all cerebellar circuits, as they are the master regulators of cerebellar development [125]. Furthermore, as Van der Heijden and Sillitoe [125] noted in their highly recommended paper, the Purkinje cell circuit has a temporal dependence on cues provided by granule cells. Granule cells are the latest born and the most populous neuronal type in the cerebellar cortex and the cells with the most NPs and structural damage as the MMC residents aged. Thus, we strongly support Van der Heijden and Sillitoe’s [125] proposal that the wiring of Purkinje cells determined by dynamically acting cues of granule cells could be at the core of the gait and equilibrium abnormalities described in MMC residents and the significant cerebellar atrophy documented in young adults by longitudinal MRI [18,126].

A striking observation documented in the current cerebellar samples and previously in the olfactory bulb [16] was the presence of endothelial erytrophagocytosis, a key element of vasopathology, according to Fens and collaborators [127]. Exposure of RBCs to oxidative stress resulted in phosphatidylserine exposure and loss of deformability and marked erythrophagocytosis in endothelial cells (ECs) under both static and flow conditions. As a consequence, EC intracellular organization was disturbed, and increased expression of apoptotic markers indicated EC cytotoxic effects. Fens and coworkers [127] concluded activated endothelial cells show significant phagocytosis of phosphatidylserine-exposing and rigid RBC. Of key importance for our vascular findings, significant erythrophagocytosis can induce endothelial cell loss. RBCs are innate NP carriers tolerating millions of NPs under experimental conditions, have low immunogenicity, great flexibility, and long systemic circulation [128]. The phosphatidylserine exposure by RBCs loaded with NPs and subjected to intense oxidative stress is a powerful signal that initiates their phagocytic removal from circulation and erythrocytes carrying NPs may enter suicidal death and eryptosis characterized by the presentation of membrane phosphatidylserine on the cell surface and cell shrinkage [129]. Excessive eryptosis may result in anemia and/or impaired microcirculation, a grim scenario for MMC residents at 2240 m above sea level exposed to significant numbers of highly reactive NPs.

The issue of nuclear pore complexes occupied by NPs and the associated structural damage deserves a comment. Nuclear pore complexes (NPCs) are grossly symmetric, gigantic molecular machines that operate as the main transportation hub between the nucleus and cytoplasm [130]. Vanneste and Van Den Bosch [130] splendidly observed that NPCs are highly selective and maintain the distinct molecular composition of the nucleus and the cytoplasm, which is very relevant to NPs: the sieve-like structure is freely permeable to objects smaller than ~30 kDa or ~5 nm, precisely the sizes of NPs we identify in the nuclear pores and traversing the nuclear membrane. Odeh et al. [131] made an important observation: alterations in nuclear-import receptors engaging nuclear-localization signals of polypeptides in the cytoplasm and transporting cargo across the size-selective barrier of the NPC into the nucleoplasm could be involved in amyotrophic lateral sclerosis (ALS), frontotemporal dementia (FTD), TDP-43 encephalopathy and tauopathies [130,131]. We strongly suggest NPs altering and obstructing NPCs ought to be researched and their causal role in nucleocytoplasmic transport alterations defined. Large detrimental cerebellar structural effects likely result in progressive damage starting in childhood; thus, cerebellar functions could be compromised as clearly described by Amore et al. [132]. The cerebellum is involved in motor functions as well as cognitive and psychological functions, including cognitive predictive functions [133,134]. The cerebellar efferent pathways have key connections with the periaqueductal gray and ventral tegmental area, non-primary motor cortex and hippocampus [133]. Moreover, the posterior cerebellum is involved in social cognition with internal action models of social interactions predicting how other people’s actions will be executed and what our most likely responses are to these actions [135]. Thus, as noted by Van Overwalle et al. [135], cognition and specifically social cognition ought to be investigated to define social dysfunctions as part of the cerebellar involvement. Indeed, early cerebellar involvement impacts specific components of executive function in childhood, adolescence, and adulthood and compromise’s executive function performance later in life [136]. Equally important in the context of air pollution, the cerebellum has high dopamine levels with midbrain dopaminergic afferents having a wide distribution of the dopaminergic receptor subtypes (DRD_1_-DRD_5_) [137] and is compromised in PD patients, as evidenced by a reduction in cerebellum WM integrity, i.e., assessed by fractional anisotropy, mean diffusivity, radial diffusivity, and axial diffusivity measures [138]. Indeed, our findings of altered NVUs in cerebellar tissues of young children raise the issue of the developing cerebellum as a target for neurotoxicants, [139] including highly reactive and magnetic NPs. The fact that the cerebellum is part of the picture in a number of neurodevelopmental and neurodegenerative diseases cannot be ignored: the cerebellum could be an early player in neurodegenerative processes as shown in FTD associated with behavioral disruption and cognitive dysfunction [38,140,141,142,143,144]. Also relevant to our MMC previous findings [145], the cerebellum significantly impacts the regulation of the sleep-wakefulness transition [146].

There were advantages and limitations to our study. A major advantage was the multidisciplinary collaboration and the efforts made to exchange viewpoints regarding the importance of emissions, portals of entry and early clinical detection of young individuals at risk, all key in understanding the impact of our findings in future multidisciplinary studies. Our major gaps were the lack of funding and the paucity of ambient NP concentration data. We needed support to extend the HRSTEM/EDX studies, and it was also extremely difficult to find clean cities with forensic labs; thus, we had limited access to clean city brain samples.

## 5. Concluding Remarks

1. The MMC neuropathological LC, SNpc and cerebellar results identify unequivocal accumulation in young residents of highly reactive and toxic NPs documented in the typical airborne MMC PM_2.5_ pollution mixtures [47,53,54,55,56,57,58,59,60,61,69,70,72].
2. The portals of entry and the specific characteristics (composition and size) of the NPs are critical in defining which cells and organelles are affected. The SN, LC and the cerebellum are early NP targets and p-τ is the most common aberrant protein ID in young urbanites [12].
3. Strikingly, we have identified in SNpc and LC neural cells and macrophages in situ NPs containing Fe, Ti, W and Hg associated with extensive NVU, mitochondrial and nuclear damage. The elongated Ti-rich NPs are similar to the ones described by our group in neuroenteric neurons, strongly supporting the gastrointestinal portal of entry is at work in young urbanites [12].
4. Iron-rich NPs in SN, LC and cerebellar children’s tissues potentially represent a severe risk; they can generate heat under an alternating magnetic field and/or magnetic field gradients, making possible particle displacement/rotation and localized heating through microwave absorption [106,107,108,109,110]. Children are extensively exposed to low frequency electric and magnetic fields (EMFs) of various frequencies and wireless networks Wi-Fi involving at least one Wi-Fi antenna using a 2.4 GHz band [147,148,149]. High-voltage power lines, transformer buildings, domestic appliances e.g., hair dryers, electric shavers, induction cookers plus compact fluorescent lamps, inductive charging systems for electric cars and security or anti-theft devices ought to be included for possible future risk analysis as clearly stated by Gajsek et al. in their Electromagnetic Field (EMF) Exposure Assessment in Europe [148].
5. The presence of HgNPs in neural cells is extremely worrisome, and the accumulation of Hg and Fe NPs in macrophage-like cells associated with LC and SNpc neurons is striking and heightens the possibility of neuronal damage by activated macrophages capable of accumulating NPs, likely through scavenger receptor-mediated endocytosis and lysosomal internalization as seen experimentally upon the administration of iron oxide nanoparticles [150].

It is also critical for the pertinent authorities to keep in mind that an NP profile with Fe, Ti, W, Hg and Zn opens the opportunity to meticulously identify the emission sources of the specific metals and in turn, making a link between early clinical alterations in exposed young populations. The bleak facts of sporadic AD and PD associated with high PM exposures is that cognitive deficits develop very early and deeply compromise the potential academic, social and economic goals in young subjects, while clinical premotor manifestations in PD are also early but unfortunately mostly ignored. NP exposure ought to be included in any assessment of the neurodegenerative risk profile of exposed individuals. No matter the entry portal, the chronic delivery of exogenous NPs to the brain induce oxidative stress and neuroinflammation [92,93,94,95,96,97]. Early life exposure to NPs poses a significant risk to brain development [7] and many current children’s studies focusing on air pollution are likely to show early NP effects [151].

We strongly suggest that highly oxidative, magnetic, metal NPs emitted in the urban atmosphere constitute a unique path to AD and PD pathogenesis deserving our full attention. Exposed children and young adults need early neuroprotection, and multidisciplinary prevention efforts must be implemented. Control of combustion and friction NP sources, and engineered NPs (food products, cosmetics, toothpaste, sun protectors, surface disinfectants, paints, e-waste, etc.) becomes increasingly important and urgent in reducing the human and economic costs of a global neurodegenerative epidemic, certainly heightened by the SARS-CoV-2 pandemic [152].

## Figures and Tables

**Table 1:** Immunohistochemistry data. Hyperphosphorylated tau and alpha-synuclein in substantia nigrae and locus coeruleus in 179 cases, average age 25.9 ± 9.2 years and 7 subjects older than 41 years old (63 ± 14.5 years, range 45–85 years).

Anatomical Area	Number of Samples	Pτ Positive IHC	α Syn + IHC	% Positive Cases Pτ/α Syn	Average Age IHC + Cases	TEM/EDX Selected Samples
Substantia nigrae (SN)	184	100	42	54.3%/22.8%	25.8 ± 9.3 years Pτ 26.7 ± 8.1 years α S	34+ and 28− for Pτ and/or α Syn
Locus coeruleus (LC)	180	82	44	44.5%/24.4%	26.7 ± 8.5 years Pτ 27.9 ± 8.2 years α S	34+ and 23− for Pτ/α Syn

## Data Availability

All data included in the paper.
